# Transcriptome profiling in conifers and the PiceaGenExpress database show patterns of diversification within gene families and interspecific conservation in vascular gene expression

**DOI:** 10.1186/1471-2164-13-434

**Published:** 2012-08-29

**Authors:** Elie Raherison, Philippe Rigault, Sébastien Caron, Pier-Luc Poulin, Brian Boyle, Jukka-Pekka Verta, Isabelle Giguère, Claude Bomal, Jörg Bohlmann, John MacKay

**Affiliations:** 1 Center for Forest Research and Institute for Integrative and Systems Biology, Université Laval, Québec, QC, Canada, G1V 0A6; 2 Gydle Inc., Québec, QC, Canada; 3 Michael Smith Laboratories, University of British-Columbia, Vancouver, BC, Canada

## Abstract

**Background:**

Conifers have very large genomes (13 to 30 Gigabases) that are mostly uncharacterized although extensive cDNA resources have recently become available. This report presents a global overview of transcriptome variation in a conifer tree and documents conservation and diversity of gene expression patterns among major vegetative tissues.

**Results:**

An oligonucleotide microarray was developed from Picea glauca and P. sitchensis cDNA datasets. It represents 23,853 unique genes and was shown to be suitable for transcriptome profiling in several species. A comparison of secondary xylem and phelloderm tissues showed that preferential expression in these vascular tissues was highly conserved among Picea spp. RNA-Sequencing strongly confirmed tissue preferential expression and provided a robust validation of the microarray design. A small database of transcription profiles called PiceaGenExpress was developed from over 150 hybridizations spanning eight major tissue types. In total, transcripts were detected for 92% of the genes on the microarray, in at least one tissue. Non-annotated genes were predominantly expressed at low levels in fewer tissues than genes of known or predicted function. Diversity of expression within gene families may be rapidly assessed from PiceaGenExpress. In conifer trees, dehydrins and late embryogenesis abundant (LEA) osmotic regulation proteins occur in large gene families compared to angiosperms. Strong contrasts and low diversity was observed in the dehydrin family, while diverse patterns suggested a greater degree of diversification among LEAs.

**Conclusion:**

Together, the oligonucleotide microarray and the PiceaGenExpress database represent the first resource of this kind for gymnosperm plants. The spruce transcriptome analysis reported here is expected to accelerate genetic studies in the large and important group comprised of conifer trees.

## Background

Microarray (MA) transcript profiling and RNA sequencing (RNA-Seq) represent powerful approaches to rapidly gain functional information on a genome-wide scale. Information on RNA transcript abundance is a key to assessing the biological role of gene products and cannot be directly deduced from a gene’s sequence. This has lead researchers to develop databases of RNA abundance profiles, first and foremost for model organisms. For example, the AtGenExpress database was created for the model-plant Arabidopsis from a host of tissue preferential and stress response expression profiles [1]. Databases such as AtGenExpress are particularly useful for the identification of groups of co-expressed genes. Other plant oriented databases include the poplar PopGenIE made up of tissue, developmental and stress response profiles [2]. Reflecting the value of gene expression data, public organizations and institutes also maintain generic databases like the Gene Expression Omnibus or GEO (NCBI) and ArrayExpress (EBI), among others, which host datasets from a wide array of organisms.

Recent transcriptome-wide analyses underscore the importance of gene expression in the genetic architecture of complex traits. Studies in fruit flies, mice, humans and maize show that a proportion of the genetic variants underlying complex phenotypes exert their effects through gene expression [3,4]; so, discovering the genetic basis for the variation in transcript abundance is central to understanding phenotypic variation [5]. Gene expression studies also provide insights into the molecular impacts of natural selection. For example, expression profiling showed the differential action of selection pressure on different tissues and organs in humans [6]. A comparative analysis of mouse and human showed a high level of conservation in the expression of orthologous genes, showing the stability of house-keeping genes and the variability of tissue specific genes [7].

Transcriptome profiling is facilitated by the availability of a reference genome but many studies have also been based on large-scale cDNA sequence datasets. In plants, many angiosperm genomes have been sequenced, including the model plant Arabidopsis thaliana [8], rice [9], poplar [10] and grapevine [11]; however, reference genomes are still lacking for plant phyla belonging to the gymnosperms. The best studied gymnosperms are conifers, which as a group have extremely large genomes (ranging from 13 to 30 Gb). In conifers including pines (Pinus spp.), spruces (Picea spp.), Douglas-fir (Pseudotsuga menziesii) and Japanese cedar (Cryptomeria japonica) over 1 million expressed sequence tags (ESTs) have been obtained from dideoxy sequencing and assembled to infer putative unigenes or transcript sets (reviewed in [12]). Large collections of cDNAs are available for white spruce (Picea glauca) [13] and Sitka spruce (P. sitchensis) [14]. From 30% to 40% of conifer sequences cannot be annotated because they lack sequence similarity to known genes [13-16].

This report describes a large-scale oligonucleotide microarray developed from the extensive cDNA datasets available for spruce trees (P. glauca, P. sitchensis) to achieve broad transcriptome coverage. Previously, microarrays were developed from PCR amplicons (cDNA microarrays) primarily in pines and spruces (reviewed in [12]). Many of the cDNA microarrays have ranged from a few hundred to several thousand cDNAs, and a few of them have included over 20,000 spots, i.e. in Picea sitchensis [17] and Pinus taeda [18]. They have essentially been used in comparative experiments (using two-dye designs) to investigate transcriptome remodeling during tissue differentiation, development, or in response to environmental cues [12], but a general characterization of conifer transcriptomes has been lacking.

A major goal of the present study was to assemble transcript profiles from spruce trees (Picea spp.) into a database called PiceaGenExpress, aiming to characterize the basic features of a conifer transcriptome such as the number of transcribed genes in a variety of tissues. The reference profiles in PiceaGenExpress enabled exploratory analyses of the diversity of expression patterns within and among gene families and the expression of retrotransposons. In addition, conservation of gene expression in secondary vascular tissue was studied based on interspecific comparisons of tissue preferential expression. The accuracy of microarray profiles and design was directly evaluated by RNA-Seq analysis of the same samples as those used for one of the microarray experiments included in PiceaGenExpress.

## Results and Discussion

### Development of an oligonucleotide microarray for spruces (Picea spp.)

A large-scale custom microarray containing 31,604 oligonucleotide probes was designed for broad representation of the spruce (Picea spp) transcriptome. The 70 nucleotide probes were based on unique cDNA sequences from white spruce (P. glauca) [13] and Sitka spruces (P. sitchensis) [14] (Table 1). Both of these conifers have extensive ESTs and FL-cDNA sequence databases developed from dideoxy sequencing (Sanger method); 454 ESTs (GS-FLX) were also available for P. glauca. The probe design parameters and microarray manufacturing methods were experimentally determined through hybridization experiments with a microarray of 3,900 oligonucleotide probes specifically designed for optimization tests (for details see Additional file 1, Additional file 2: Figure S1 and Additional file 3: Figure S2).

**Table 1:** Development a large-scale oligonucleotide array for spruces (*Picea spp*): sequence information used to design oligonucleotide probes from *Picea glauca *and *P. sitchensis *sequences

**Category**	**Number of probes**	**%**	**Probe designed**^**1**^	**Confirmation of *P. glauca (Pgl) *cDNA clone or other**
1	11,214	35%	Pgl	Confirmed in Pgl and Psi cDNAs
2	12,251	39%	Pgl	Confirmed by Pgl 454 seqs or Psi cDNAs
3	4,840	15%	Psi	Confirmed by Pgl 454 seq
4	1,629	5%	Pgl	Unconfirmed but Pgl clones ≥ 2
5	1,670	5%	Psi	Psi full-length cDNA only (not found *Pgl*)
All	31,604			

^1^Each probe was designed based on the sequence of *P. glauca* (Pgl) or *P. Sitchensis* (Psi).

The high level of sequence identity in the Picea datasets enabled the design of a single probe matching both P. glauca and P. sitchensis sequences, for most genes; however, the datasets did not overlap entirely so that some probes were unique to one of the species and could not be verified in the other. A level of 95.7% of sequence identity or higher (3 mismatches or less) was obtained for nearly all of the probes relative to P. glauca sequences, and for 59% of the probes relative to P. sitchensis (Table 2). Preliminary experiments indicated that 3 mismatches had a small impact on the hybridization signal intensity and the ability to detect differential expression (See Additional file 1, Additional file 2: Figure S1 and Additional file 3: Figure S2). Analyses presented in this report are based on the set of 25,045 probes designed from P. glauca sequences, which match 23,853 unique cDNAs in the P. glauca gene catalogue of Rigault et al. [13].

**Table 2:** Analysis of probes: sequence similarity of probes aligned to *Picea glauca *and *P. sitchensis *sequences

***P. glauca***	***P. sitchensis***	**Number of probes**	**%**
67-70 (>95.7%)	67-70 (>95.7%)	17,279	54%
67-70 (>95.7%)	NA	11,999	38%
67-70 (>95.7%)	63-66 (>90%;<95%)	923	3%
63-66 (>90%;<95%)	67-70 (>95.7%)	529	2%
NA	67-70 (>95%)	869	3%

**Figure 1:**
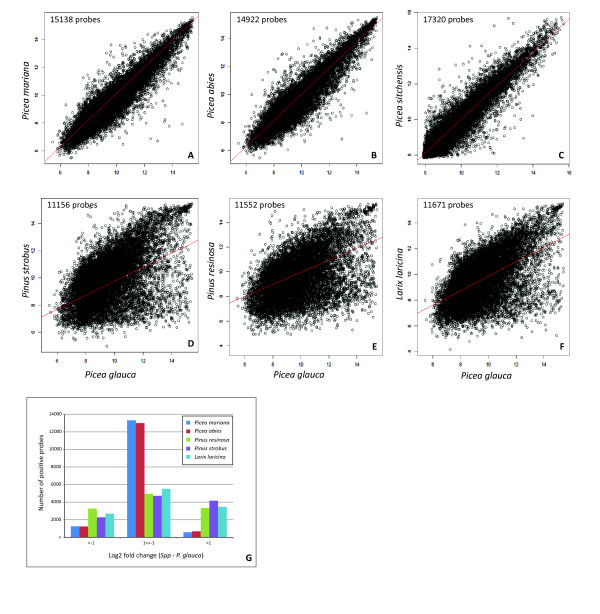
**Interspecific comparison of hybridization intensities in secondary xylem.A-F**: Pair-wise comparison of white spruce and six other species based on the number of shared positive probes indicated in the plots. The squared correlation coefficients (r^2^) are as follows 0.86 (**A**), 0.85 (**B**), 0.89 (**C**), 0.29 (**D**), 0.22 (**E**) and 0.27 (**F**). **G**: Analysis of signal intensity variation between species; the fold change (FC) was determined from the average normalized signal intensities (log2 scale). An FC of 1 or −1 represents a two-fold signal increase or decrease, respectively. For phelloderm results, see Additional file 4: Figure S3.

The potential for utilizing this oligonucleotide microarray in other species and genera of the Pinaceae family was evaluated by using comparative RNA hybridizations with four different spruces (Picea spp), two pines (Pinus spp.) and a larch (Larix laricina) (Figure 1 and Additional file 4: Figure S3). The hybridization outcomes were highly similar among the spruces (Figure 1A-1C). A large majority of the probe signal intensities varied less than 2-fold among the spruces (Figure 1G). When compared to P. glauca, the pines and larch gave more divergent results as shown by the number of common positive probes (above background) and overall data correlations (Figure 1D-1F). Surprisingly, the pines and larch gave signals of equal or greater intensity than P. glauca for most of the probes, and a decreased signal for 17% to 34% of the detected probes. Taken together, these observations indicated that the microarray is suitable for direct comparisons of transcript levels between spruces. For pines and larch, a subset of the probes may not be informative. In general, the MA appears appropriate for studies comparing data within the same genus.

### Differential expression in the vascular transcriptome is conserved among Picea species

**Figure 2:**
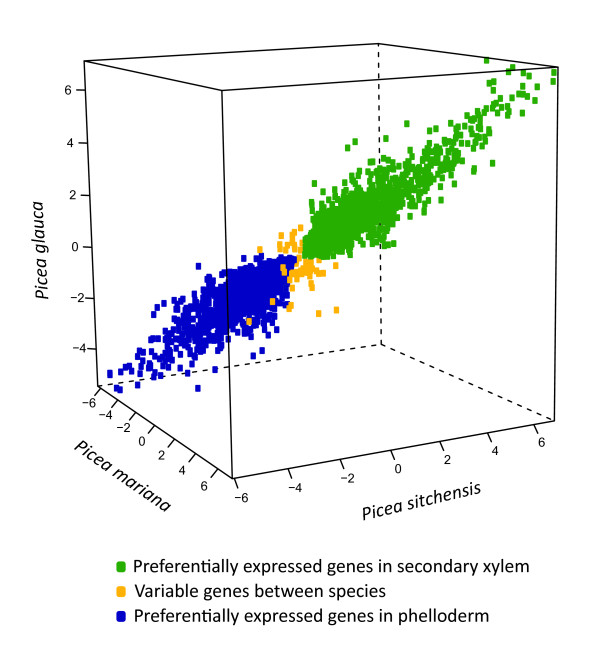
**Preferential expression in secondary vascular tissues of three spruce species.** The FC data in the plot represent genes with differential expression in all three spruces. The scale is the log2 fold change.

Microarray transcript profiles compared two tissues that support secondary vascular growth, i.e. diameter stem growth, as it is a key feature of the life habit of trees. Secondary xylem is the wood forming tissue located on the internal side of the cambial meristem. It was compared to a composite sample of phloem and phelloderm tissues (referred to here as phelloderm) located on the outer side of the cambial meristem in three spruce species. Identical analyses were carried out in three spruces: P. glauca, P. sitchensis and P. mariana. The number of transcripts detected for these two tissue types were highly conserved in the three species, ranging from 13,744 to 14,513 in xylem, and 14,990 to 15,697 in phelloderm. A total of 5,407 genes were differentially expressed (DE) in all three species. Tissue preferential transcript accumulation and the fold difference between the tissues were very similar among the three species (Figure 2). The small number of genes that varied in their tissue specificity (60 genes or 1.1% of the DE genes) indicated that genes with small difference in expression between tissues were more prone to vary between species or be less accurately determined.

Different MA experiments comparing xylem and phloem tissues in angiosperms including Arabidopsis [19,20] and poplar [21], and in P. glauca have helped to delineate groups of genes whose expression was of particular relevance to secondary vascular growth. A MA profiling study in Arabidopsis root-hypocotyl defined a set of 319 genes specifically regulated in secondary xylem compared to phloem or non-vascular tissues [19]. In young spruce trees, 360 sequences were shown to be xylem preferential compared to needles and phloem [22]. A core set of 52 xylem genes was identified by Ko et al. [21] based on transcriptome analyses of secondary xylem in Arabidopsis thaliana and poplar, and of cotton fibres. The expression patterns reported here for three spruces indicated that tissue preferential expression for xylem compared to phelloderm were conserved among spruces. These conserved patterns could be the basis for comparative genomics of conifers and angiosperms trees. This finding is also relevant for studying the genetic architecture of wood traits because it was shown that xylem preferential expression was a feature of genes associated with genetic variation in wood properties [23].

### Validation of the microarray and profiling results by RNA-Sequencing

An RNA-Seq study P. glauca secondary xylem and phelloderm used the same RNA samples as for the MA profiling, but the samples for each tissue type were pooled before sequencing. A total of 59.5 M high-quality sequences were obtained and mapped to the 27,720 cDNA clusters of the P. glauca gene catalogue as previously described [13] (Table 3). The sequence frequency data were normalized by transforming the data to reads per kilobase of exon model per million mapped reads (RPKM) as described by Mortazavi et al. [24]. These authors estimated that an RKPM > 1 represents one RNA molecule per cell; therefore, an RPKM > 1 was used as a threshold for detection.

**Table 3:** RNA-Seq data

	**Xylem**	**Phelloderm**	**Total or Both**
Total reads (HQ) (millions)	29.5	29.9	59.5
Reads mapped (millions)	17.3	20.4	37.7
Genes - RPKM^1^ > 1	19,604	21,366	22,012
Genes - RPKM > 3	16,168	18,297	19,108

^1^ RPKM, Number of reads per kilobase of mapped sequences per megabase based on Mortazavi et al. [24].

Nearly all of the DE genes from the MA analysis (99%) were represented in at least one of the RNA-Seq samples, and a large majority (84% to 88%) of genes with a 2-fold difference on the MA, were also differentially represented in RNA-Seq (Table 4). These RNA-Seq data confirmed the tissue preferential expression (xylem vs. phelloderm) of 99.3% of the genes determined to be differentially expressed with both methods (Table 4). The cross-validations between the two methods were based on differential expression in 2,171 to 4,181 genes depending on the fold difference threshold; therefore, it represented a highly robust confirmation of the accuracy of the MA results. The overlap in the number of genes varied depending on the fold difference threshold, likely owing to differences in experimental design and technique; however, the preferential expression (xylem vs. phelloderm) was highly congruent between the analysis methods (Table 4).

**Table 4:** Validation of microarray results by RNA-Seq

**Criteria**		**MA**^**1**^	**RNA-Seq and MA**^**2**^						**RNA-Seq only**^**4**^	
**P-value**	**FC (log2)**		**RPKM > 1**		**DE**^**3**^		**Tissue pref.**		**DE**	**Add**
0.05	0.5	5,666	5,592	99%	4,181	75%	4,155	99%	776	19%
0.05	1.0	2,614	2,588	99%	2,265	88%	2,253	99%	677	30%
0.01	0.5	5,526	5,466	99%	3,608	66%	3,585	99%	542	15%
0.01	1.0	2,608	2,582	99%	2,171	84%	2,160	99%	491	23%

^1^ DE positive genes determined by MA.

^2^ Number of DE genes detected by RNA-Seq among the DE genes found by MA hybridization; Tissue pref., indicates that the tissue preference for xylem or phelloderm was the same.

^3^ DE, differential expression in RNA-Seq, determined by a Chi-squared test (df = 1) with Benjamini-Hochberg correction for multiple testing.

^4^ RNA-Seq results for non-DE genes from MA (considering genes represented on the MA only).

The RNA-Seq detected more transcribed genes than the MA analysis, i.e. close to 6,000 genes with an RPKM > 1 (Table 4). The number of DE genes determined by RNA-Seq and not by MA ranged from 491 to 776 (Table 4), although these results are only suggestive given the experimental design used for the RNA-Seq. In addition, some of the genes shown to be tissue preferential by MA analysis were suggested by RNA-Seq to be specifically expressed only in one of the tissue types (Table 5). Interestingly, 50% to 65% of these putative tissue-specific sequences had no annotation based on similarity to TAIR sequences or the detection of Pfam domains, compared to, less than 40% for the entire set of genes represented on the microarray [13]. This observation is consistent with findings from animal research showing that genes that have more specific expression patterns also tend to have less conserved sequences among species [4]. In evolutionary terms, genes that are expressed only in xylem or in phloem may either represent sequences that are unique or are more highly diverged in conifers compared to other plants.

**Table 5:** Tissue specificity in RNA-Seq

	**Xylem**	**Specific**	**Phelloderm**	**Specific**
	**Total**	**Annotated**	**Total**	**Annotated**
Genes RPKM > 1	44	16 (36%)	186	79 (42%)
Genes RPKM > 3	17	6 (35%)	81	41 (50%)

### PiceaGenExpress contains reference profiles that reveal patterns of tissue preferential expression

The PiceaGenExpress database was developed from over 150 MA hybridizations of Picea spp obtained for eight sample types representing different tissues and experiments (Table 6) (for procedures, see methods). Detailed analysis of each of the eight datasets will be presented elsewhere. In any given sample type, transcripts were detected for 10,067 to 17,070 genes (hybridization signal above background threshold); transcripts were detected for 21,939 different genes considering all of the tissues, i.e. 92% of the P. glauca genes represented on the microarray. The PiceaGenExpress is made available as a flat file (Additional file 5: Table S1) for ease of upload and access. Less than 10% of the genes were unique to one sample type. A simple ranking procedure was applied, as a means to represent the relative expression and enable qualitative comparisons across samples. The genes were ranked within each dataset of PiceaGenExpress separately, based on average signal intensity in a sample type, and then equally divided into 10 intensity categories (expression classes): from the lowest 10% (class 1) to the highest 10% (class 10) (Additional file 6: Table S2, Additional file 7: Figure S4).

**Table 6:** The PiceaGenExpress database: sample characteristics, hybridizations and detected genes

	**Plant material**			**Microarrays**			**Genes**^**5**^		
	** Tissue type**	**Sp**^**1**^	**Source sampling**^**2**^	**Genotype**^**3**^	**Slide**	**Imaging**^**4**^	**Total**	**Unique**	**Non-annotated**
1	Embryogenic cells	Pgl	a, a	1	6	SQ	10,066	100	2,456
2	Vegetative buds	Pgl	b, c	2	10	SQ	12,361	128	3,216
3	Xylem (Mature)	Pgl	d, d	60	60	SQ	14,686	176	4,232
4	Xylem (juvenile)	Pgl	e, f	30	20	SQ	13,807	56	3,701
5	Phelloderm	Pgl	e, f	30	20	SQ	15,803	214	4,391
6	Young needles	Pgl	e, f	30	10	SQ	12,819	167	3,025
7	Megagametophytes	Pgl	g, c	3	3	PA	17,056	1,111	5,205
8	Adventitious roots	Pab	h, c	8	20	PA	15,718	393	4,696
					Total detected		21,241	2,345	
					Not detected		2,612		

^1^ Pgl: Picea glauca (White spruce); Pab: Picea abies (Norway spruce).

^2^ The source of materials and the sampling method are from the following studies: a, [43]; b, [37]; c, this paper, see methods; d, [23]; e, O-P seedlot, this paper see methods; f, [22]; g, C2856 parent from [38]; h, [44].

^3^ Total numbers of genotypes analyzed (either individually or in pools).

^4^ Microarray Scanner and Image Processing: SQ, ScanArray Express (Perkin Elmer, Waltham MA, USA) and QuantArray v3.0 (Packard BioChip Technologies, Billerica, MA, USA); PA, PowerScanner (Tecan Group Ltd., Männedorf, Switzerland) and ArrayPro Analyser v6.3 (Media Cybernetics, Bethesda, MD, USA).

^5^ Total: number of genes above background (see methods); Unique: genes detected only in one tissue.

**Figure 3:**
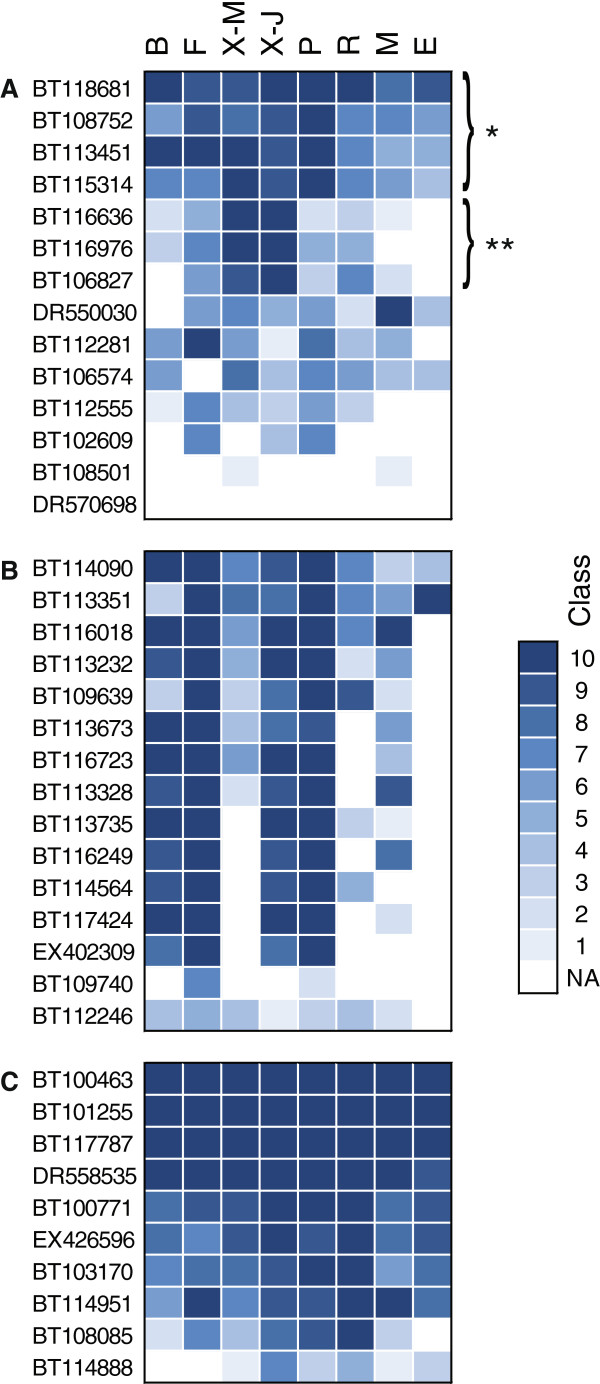
**The PiceaGenExpress database reveals tissue preferential and conserved expression patterns within three gene families.A**: Cellulose synthases. **B**: Photosystem I and II proteins. **C**: Ubiquitins. NA: Not detected. Tissues: B (Vegetative buds), F (Foliage), X-M (Xylem - mature), X-J (Xylem - juvenile), P (Phelloderm), R (Adventitious roots), M (Megagametophytes), E (Embryogenic cells).

The PiceaGenExpress database was used as a tool to rapidly assess tissue preferential and invariant expression profiles (Figure 3). It allowed us to identify gene families with diverse patterns of tissue preferential expression. For example, transcripts for three cellulose synthase (CS) genes stood out as being strongly overrepresented in the secondary xylem (both juvenile and mature) compared to all other tissues, where as other CS transcripts were more ubiquitous (Figure 3A). This observation was consistent with studies in pine [25] and poplar [26] showing that CesA genes that are specialized in secondary cell wall formation occurred as triplets of genes. A different type of pattern was observed with transcripts for photosystem I and II proteins, which are generally strongly expressed in green tissues (Figure 3B). As might be expected, transcripts of all of the genes in this class were detected at high levels and were co-expressed in young needles, expanding buds, and phelloderm of young trees, and at low levels in non-photosynthetic tissues including roots, immature embryos and megagametophytes. For secondary xylem, the same transcripts were abundant in young trees but low in the mature trees, which could be accounted for by differences in exposure to light and bark thickness. Genes encoding house-keeping proteins like ubiquitin did not vary between the tissues (Figure 3C). These observations relating to tissue preferential expression are rapid and simple within PiceaGenExpress. They are also consistent with known expression and physiology, and suggest that the methodology that was followed to develop the database is adequate for revealing key patterns of gene expression.

### Non-annotated genes are expressed at low levels and in fewer tissues

**Figure 4:**
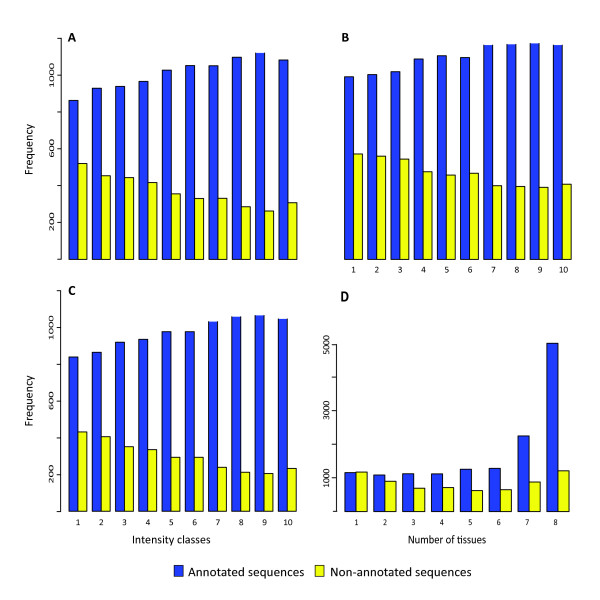
**Expression classes and numbers of tissue of annotated and non annotated sequences.A-C**: Number of annotated and nonannotated sequences per expression class for xylem from juvenile trees (**A**), roots (**B**) and young foliage (**C**). Other tissues are shown in Additional file 8: Figure S5. **D**: Number of tissues in which each annotated and non-annotated sequence was detected. Frequency, number of genes in a given intensity class or detected in a given number of tissues types.

From 30% to 40% of cDNAs from non-model organisms such as conifers could not be annotated by standard sequence similarity searches like BLAST [15,16] or HMMER [13]. We investigated whether insights into the role of non-annotated sequences could be obtained by surveying their abundance and distribution among tissues in PiceaGenExpress (Figure 4; Additional file 8: Figure S5). First, we observed that non-annotated sequences, i.e. sequences that lacked similarity to known plant genes, were more represented among low abundance transcript classes. On average, the non-annotated sequences represented 37.1% of the expression class 1 genes and 22.4% in class 10 (not shown), and a same trend was observed in each of the tissue types (Figure 4A-C; Additional file 8: Figure S5A-E). These non-annotated sequences could be either unique to conifers, owing to differential loss or acquisition of genes among taxa, or could be too highly diverged to permit functional annotation. Their general low level of expression may suggest that they were expressed in fewer cells or were tightly regulated in some manner, perhaps playing a more specialized role in metabolism or development.

Second, our data showed that annotated and non-annotated genes were strongly contrasted in regard to the number of tissues in which transcripts were detected (Figure 4D). The majority of the annotated sequences were detected in seven or eight of the tissues. In contrast, the non-annotated genes were much more likely to be found in few tissues. This observation is consistent with the idea that less conserved (including non-annotated) sequences may generally play more specialized roles. It is also consistent with findings from comparative expression studies of mice and humans showing that house-keeping genes were more highly expressed and were more conserved among species both in terms of their sequence and their expression [7].

### Diversity of expression profiles varies within and among gene families with related functions

**Figure 5:**
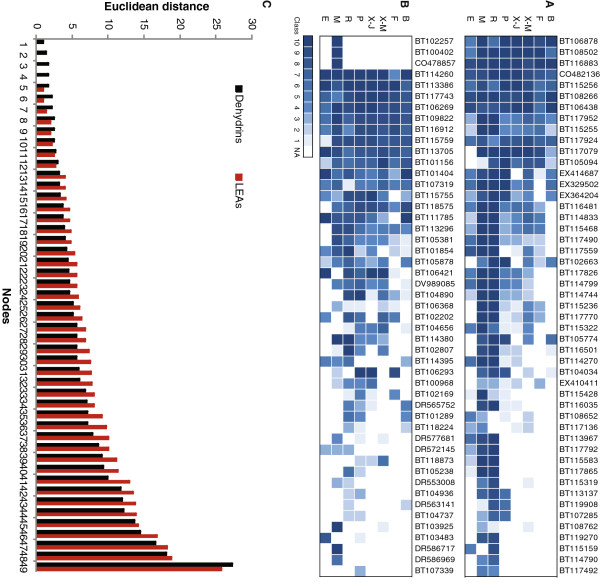
**Gene expression patterns in two osmotic regulation protein families based on the PiceaGenExpress database.A**: Dehydrins. **B**: Late Embryogenesis Abundant proteins. **C**: Distribution of Euclidean distances between the members of each protein family. The order of the bars is not representative of the order of the genes in panels A and B. A, B: Tissues: B (Vegetative buds), F (Foliage), X-M (Xylem - mature), X-J (Xylem - juvenile), P (Phelloderm), R (Adventitious roots), M (Megagametophytes), E (Embryogenic cells).

A total of 28 different gene families were reported to be statistically overrepresented in spruce compared to major angiosperms based on the occurrence of protein domains of known function [13]. Approximately one fifth of them were related to stress responses including osmotic regulation proteins like dehydrin and late embryogenesis abundant protein (LEA) gene families. The two families are of similar size in the white spruce gene catalogue, with 49 sequences each [13]. Expression profiles (distribution of transcript abundance classes) within these families were examined in PiceaGenExpress to gain insight into the extent of functional divergence or redundancy that might be associated with gene family expansion. The expression of 49 different sequences containing a dehydrin protein domain indicated striking differences between tissue types during normal development. Many more dehydrin sequences were detected in roots (high expression classes), megagametophytes and, to some extent, in the phelloderm than in the other tissues (Figure 5A). A large group of the sequences seemed to be co-expressed, i.e. all were expressed either very strongly (class 9–10) or more weakly (class < 5).

Expression profiles of the 49 gene sequences containing a LEA domain also varied between tissues and appeared more diversified than observed for dehydrins (Figure 5B). The extent of divergence among the members in each family was analyzed by clustering and determination of Euclidean distances among the sequences (Figure 5C). Overall most of the nodes (38 out of 49) were separated by a greater distance in the LEA family than in the dehydrin family, indicating that during normal development, the regulation of the dehydrin family members is less diversified. This observation may point at greater functional diversification among LEAs.

Both dehydrins and LEAs have been shown to be expressed during normal development and to be water stress responsive in conifers [18,27,28]. Our study only considered expression during normal development. Previous studies in conifers have monitored the expression of a small subset of these large gene families. For example, five and eight dehydrins transcript sequences were studied in foliage of maritime pine [27] and vegetative buds of Norway spruce, respectively [28]. We detected 22 distinct sequences by MA profiling in young foliage, strongly suggesting that a comprehensive view of this protein family in response to stress remains to be developed. The expression profiles presented here indicated that osmotic-regulation during normal development may involve more genes (especially dehydrin genes) and potentially more varied functions in some of the tissues (megagametophytes, roots and phelloderm) than others (foliage, embryos). This observation indicates that osmotic response monitoring, whether it is related to drought conditions [27] or to normal developmental processes [28] may be sensitive to tissue preference. In the PiceaGenExpress dataset, several dehydrins were down regulated in vegetative buds that were sampled at the time of bud flush in the spring, as was observed through detailed time series analyses of Norway spruce dehydrins [28]. An interesting feature of the data is that the two seed derived tissues, i.e. immature somatic embryos and megagametophytes from germinating seeds, were highly contrasted in regard to the expression of specific dehydrin and LEA sequences.

### Expression of LTR retrotransposon sequences

**Figure 6:**
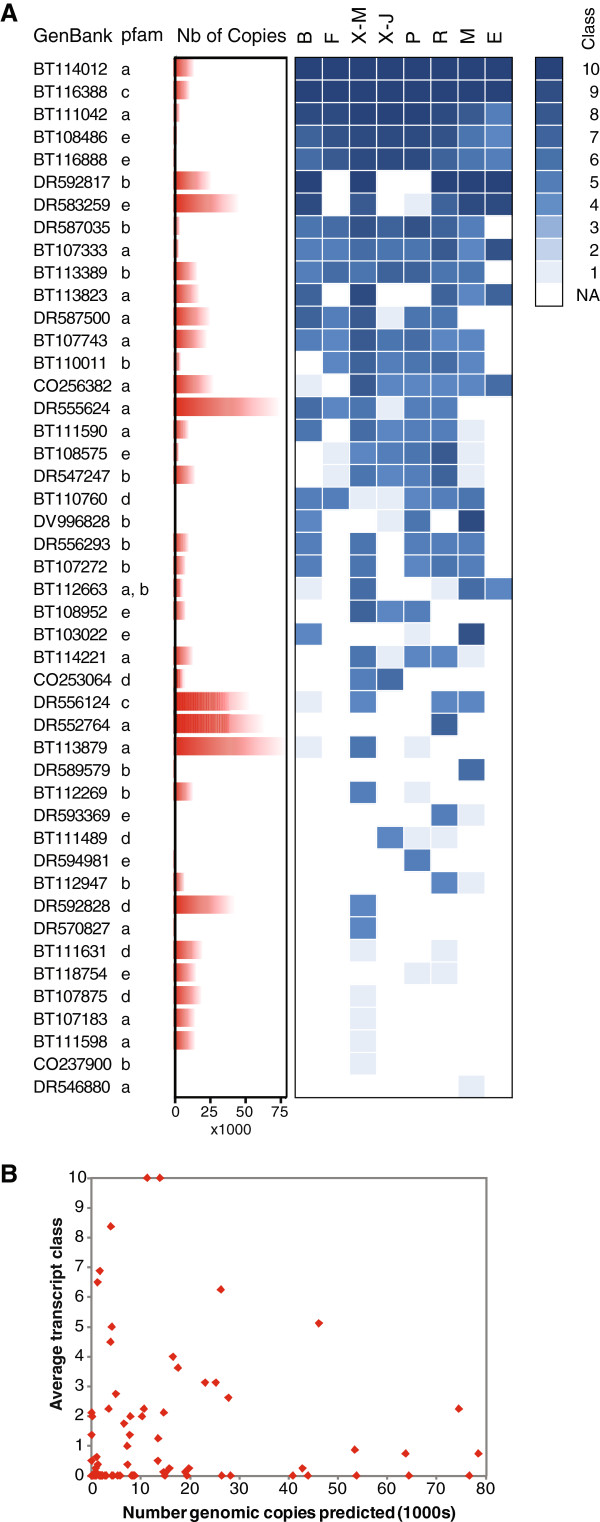
**Expression patterns of LTR retrotransposons based on the PiceaGenExpress database.A**: Sequences containing protein domains of LTR retroelements. Pfam annotations: a: Integrase core domain; b: Retrotransposon gag protein; c: Retroviral aspartyl protease; d: Reverse transcriptase (RNA-dependent DNA polymerase); e: RNAse. Tissues: B (Vegetative buds), F (Foliage), X-M (Xylem - mature), X-J (Xylem - juvenile), P (Phelloderm), R (Adventitious roots), M (Megagametophytes), E (Embryogenic cells). **B**: Expression levels are not correlated with the number of genomic copies.

Recent reports indicated that LTR retrotransposons represented a large fraction of the conifer genome [29-31]. In addition, sequences traced to copia and gypsy-like retroelements were reported as being very frequent in Pinus contorta ESTs [32] and were overrepresented in a root xylem cDNA library [33], although some of these sequences may represent genomic contaminations [13]. More evidence is needed to show whether any of these elements are active or if activity may vary as a function of development or environmental stresses. The PiceaGenExpress database was scanned to obtain evidence of RNA transcript production, which is the first step for LTR retrotransposon mobilization. A total of 83 cDNA sequences represented on the MA coded only for protein domains expected for LTR retroelements, such as RNAse H, integrase core domain, retrotransposon gag protein and reverse transcriptase. Many of the sequences were not detected at all; however, 46 sequences were detected in at least one tissue, most of them were found in two tissues or more, and only a few accumulated at high levels (expression class 10) (Figure 6A). Tissue preferential accumulation was suggested by the fact that few sequences were detected in immature somatic embryos and were present at low levels, where as many sequences were detected and in higher expression classes in mature xylem, roots and megagametophytes.

The MA sequences matching putative LTR were estimated to represent up to 78,520 unique copies in the P. glauca genome (Figure 6B) and a total of 1.18 Million sequences (not shown) based on their occurrence in shotgun sample sequencing data for P. glauca [13]. Data are not currently available to estimate what proportion of the genome these sequences may represent; however, if the sequences were derived from an intact LTR of 4000 bp on average, they would represent nearly 5 Gbp or 20% of the P. glauca genome. In other words, such a large number of copies is expected to occupy a sizable fraction of the genome. The number of predicted copies did not appear to correlate with transcript accumulation, i.e. number of tissues in which they were detected or relative levels (Figure 6A, see right panel; 6B). This observation indicates that the data were more likely to result from transcription than genomic contaminations of the RNA samples. It also shows that the number of copies accumulated in the genome is not useful for predicting the level of transcript production. In fact, many of the high copy sequences (>30,000 copies) were not detected at all in any of the tissues. These results point to specific LTR sequences that have the highest potential for mobilization in P. glauca.

## Conclusions

An oligonucleotide microarray was developed from P. glauca and P. sitchensis datasets. It represents 23,853 unique P. glauca genes or 85% of the recently reported gene catalogue [13]. Single dye analysis and a ranking procedure were used to develop PiceaGenExpress, a database of reference transcript profiles, based on 150 hybridizations in eight different tissue sample types. These data represent the first resource of this kind for a gymnosperm plant.

The pine family comprises over two hundred species belonging to eight genera. It is the largest and the most economically important of the conifers. Interspecific comparison experiments presented in this report indicated that the microarray may be applied to at least three of these genera. It could be a valuable tool for species where cDNA resources are lacking or underdeveloped. Our findings also indicate that expression profiles from P. glauca are likely to be representative of other conifers. RNA-sequencing has become a method of choice for transcriptome profiling but the analysis of RNA-Seq data can be complex owing to factors such as sequence polymorphisms, gene paralogs, and alternate splicing. Therefore, successful application of RNA-Seq depends on the availability or the development of a good quality reference genome or gene catalogue [24], and both are lacking in many non-model species. By mapping P. glauca RNA-Seq and microarray data back to the same cDNAs sequences, we were able to show that the two methods were highly congruent. New sequencing technologies promise to generate high quality sequences in addition to very large volumes of data. Given the large size of conifer genomes, it may be advantageous to use these technologies to develop high quality gene catalogues rather than attempt to assemble entire genomes. Once a gene catalogue is produced, these high throughput sequences represent a powerful methodology for unrestricted gene expression studies [24].

Conifer genomes have a number of characteristics that make them unique, most prominently their enormous size which can reach or even exceed 30 Gbp [30] and their highly repetitive sequences [31]. They are also known to have high levels of heterozygocity and are believed to harbour many gene paralogs, at least in some gene families [12]. For the MA described here, the empirical tests of probe specificity (Additional file 2: Figure S1) and the probe design parameters appeared sufficient to discriminate between paralogs that are known in the P. glauca catalogue of 27,720 genes [13]. The very high level of congruence observed between MA and RNA-Seq results, 99.3% of confirmation of tissue preference from over 2100 genes, also suggest that the MA results accurately reflect the expression of the target sequences. In contrast, issues of cross-hybridization between gene paralogs with different expression patterns would have likely resulted in a lower validation rate.

The tools and methods presented in this report may lead to diverse applications for fundamental discovery in forest genetics and evolutionary biology, such as understanding phenotypic variation in economic and adaptive traits. The genetic architecture of complex phenotypes in plants and trees is routinely probed by scanning the genome for DNA sequence polymorphisms through QTL mapping and association studies [34]. However, Huang et al. [5] summarized several recent studies by stating that discovering the genetic basis for the variation in transcript abundance was central to understanding phenotypic variation. Examining the genetic architecture of gene expression can provide functional insights into physiology and metabolism, for example by revealing the organization of gene networks [35,36].

## Methods

### Evaluation of array design and manufacture parameters with test oligonucleotide microarray

A custom MA comprised of 3,900 oligonucleotide probes was developed to evaluate the impact of design parameters (for details see Additional file 1, Additional file 2: Figure S1 and Additional file 3: Figure S2). It contained multiple probes for 929 distinct genes as well as cDNA amplicons for 96 of those genes. The parameters tested include oligonucleotide lengths of 50, 60 and 70 nucleotides, the position of probes within the transcript, the impact of SNPs and indels. The presence of one or three SNP mismatches (distributed throughout the oligonucleotide probes) had a small effect on hybridization signal intensities and in many cases the expression ratio between tissues was largely conserved (See Additional file 2: Figure S1). In contrast, the presence of seven SNP mismatches in the probe had a large effect on intensities and expression ratios. Based on these data, 70-mer probes that vary by up to three SNPs distributed throughout the probe (95.7% sequence identity) are expected to hybridize to the same RNA and on average produce a signal of similar strength, where as probes with seven SNPs or more (less than 90% sequence identity) give very little cross hybridization. These observations establish thresholds of sensitivity to sequence variation, i.e. up to three mismatches have little impact on sensitivity, and specificity, i.e. specificity is achieved with seven mismatches or more to other sequences. The cut-off is situated between four and six SNPs, i.e. between 95% and 91% sequence identity. The presence of short insertions and deletions (up to six nucleotides) located at the center of the 70-mer probes had a small impact on probe performance. The impacts of other parameters tested were generally small and less predictable.

The impact of different spotting buffers and surface chemistries used to manufacture the microarray were also assessed in regard to image quality and data reproducibility. For details on methods and findings, see Additional file 1. We found that the optimum conditions were obtained by using aminosilane coated slides and 3X SSC without betaine as a spotting buffer.

### Microarray design and manufacture

The sequences included in the microarray were selected on the basis of reproducible sequence quality from all of the ESTs and FL-cDNA described for P. glauca in Rigault et al. [13] and P. sitchensis in Ralph et al. [14] (Table 1). To obtain a robust probe set, we selected sequences that were either detected in the two species, verified with two technologies (Sanger and 454) or were derived from a FL-cDNA (Table 1). The probes were 70 nucleotides in length, and were designed to minimize similarity with other sequences in the dataset. Sequence similarity between the probes and known sequences in P. sitchensis and P. glauca was determined from sequence alignments. The microarray contains 25,045 probes that match with known P. glauca sequences; however, they represent 23,853 unique genes based on the most recent clustering [13], such that 1,017 genes are represented by more than one probe.

The microarray consists of 33,984 spotted features including 33,024 sample spots (Invitrogen, Carlsbad, CA, USA), 240 negative buffer spots, and 480 Spot Report Alien Oligos (Agilent Technologies, Inc., Santa Clara, CA, USA). Oligonucleotides were consolidated into 384-well plates, lyophilized by speed-Vac, and resuspended in 3X SSC to a printing concentration of 30 μM. Oligos were printed on aminosilane slides (Erie, Hudson, NH, USA) with a QArrayMax microarray printer (Genetix Limited, Hampshire, UK) using 946MP2 microarray pins (ArrayIt Corp, Sunnyvale, CA, USA) in a 48-pin tool depositing ~0.5 nL per spot onto the slide. The resulting microarrays had a 4 x 12 subgrid layout with 708 spots per subgrid, each spot having approximate diameter and pitch of 90 μm and 160 μm, respectively. A 280-bp GFP (green fluorescent protein) oligonucleotide was printed in subgrid corners to assist in grid alignment during image processing. The slides were crosslinked in a UV Stratalinker 2400 (Agilent Technologies, Inc.) at 300 mJ. Array quality was assessed by visual inspection and hybridization of representative slides from a print run by dye-labeled random 9-mer oligonucleotides. The quality control images were acquired via the GenePix 4200AL scanner (Molecular Devices, CA, USA) at a 10 micron resolution and quantified with the Imagene 8.0 software suite. Oligonucleotide library management, printing of microarrays and quality control was performed by the Genome BC Microarray Platform (Vancouver, BC, Canada).

The array design details are available in the Gene Expression Omnibus (accession No. GPL15033). All of the transcript profiling data is also deposited in GEO (available upon acceptance of this manuscript for publication).

### Plant materials

The origin, genotypes and method of collection of each type of material are described in Table 6, except for details described here. All of the tissue samples were frozen in liquid nitrogen immediately after removal from the trees, the seed or tissue culture vessels, and stored at −80°C until further use.

•Vegetative Buds: Buds were collected from branches of several clonal replicates of 9-year-old trees of P. glauca regenerated from two genetically distinct somatic embryogenesis lines as described [37] during the mid-Spring when the buds were just beginning to grow. For each genotype, five biological samples each consisting of 6 buds (approximately 80 mg fresh weight) were used for analysis.

•Secondary xylem, phelloderm (including phloem), young needles of juvenile trees: Nursery planting stock (from open-pollinated seed lots) were obtained as 3-year-old seedlings of Picea glauca, Picea mariana, Picea abies, Picea Sitchensis, Pinus strobus, Pinus resinosa and Larix laricina, were transferred to 8-inch pots and grown in a greenhouse under natural light conditions. Sampling of tissues was timed with the ending of primary shoot elongation, i.e. after 6 to 8 weeks of growth, and was as described [22]. For each tissue type five biological samples were prepared by pooling 6 independent trees within each species. These materials were used for interspecific comparisons (all 6 species) and for the development of PiceaGenExpress (P. glauca only).

•Megagametophytes: Control-pollinated seed (cross C962856) were obtained from P. glauca tree (80112) described in [38], were surface-sterilized for 1 minute in 70% EtOH and 10 minutes in 3% Na-hypochlorite, washing the seeds with sterile water three times between and after treatments. The seeds were then immersed in 4°C sterile water for 24 hours and stratified at 4°C for 28 days. Next, the seeds were moved to 26°C on petri dishes with a moist paper and kept in the dark to start the germination process. After 4 hours of incubation the seeds were opened and the embryo was separated from the megagametophyte under a dissecting microscope. A total of 3 biological samples comprised of a single megagametophyte were used for analysis.

•Adventitious roots: Norway spruce (P. abies) seedlings were grown in the experimental nursery of Finnish Forest Research Institute for about one and a half years before sampling. They were grown in standard nursery growing media, light Sphagnum peat, and fertilized with mineral nutrients. Roots were washed under tap water to remove surrounding peat and approximately 1 cm of the root ends was collected for analysis. Between two and four biological samples comprised of several root ends were analyzed for each of eight different genotypes.

### RNA extraction, labelling and hybridization

Total RNA was extracted following Chang et al. [39] as described in Pavy et al. [22] for all of the sample types, except for megagametophytes where poly A + RNA was extracted directly by using polyT coated magnetic beads (Dynal). One μg of total RNA was amplified for each sample replicates, except for the megagametophyte samples where 10 ng of poly A + RNA was used, with the Amino Allyl MessageAmp II aRNA Amplification Kit (Applied Biosystems by Life Technologies, Carlsbad, CA, USA) according to manufacturer’s instructions. Five μg of amplified RNA (aRNA) was then labelled with Alexa Fluor 555 or 647 dyes (Invitrogen, Carlsbad, CA, USA) and purified as per the manufacturer’s instructions. Dye incorporation efficiency was determined by using a Nanodrop spectrophotometer (Thermo Fisher Scientific, Waltham, MA, USA) following the manufacturer’s instructions. Depending on the experiment, each microarray was hybridized with one labeled aRNA sample or two samples labelled with different dyes. The sample(s) to be hybridized to a microarray were mixed and the volume was reduced to ~10 μl by evaporating excess water in a DNA 120 speedvac (Thermo Fisher Scientific). Labelled aRNAs were fragmented for 15 minutes at 70°C using Ambion’s ”RNA Fragmentation Reagents“ (Applied Biosystems), placed on ice for 1 minute, denatured for 2 minutes at 95°C, put on ice for 2 min and resuspended in 120 μl hybridization buffer (50% formamide, 5X SSC, 0,1% SDS, 0,1 mg/mL Herring sperm DNA) preheated to 55°C. Samples were kept in a heating block at 50°C until hybridization.

Hybridizations were performed in HS400Pro hybridization stations (Tecan Group Ltd., Männedorf, Switzerland). The slides were heated at 80°C for 10 minutes, then washed once at 37°C with 0.5X SSC, 0.1% SDS for 20 seconds and once at 50°C with 2X SSC, 0.5% SDS for 20 seconds, and prehybridized for 1 hour at 65°C in 5X SSC, 0.1% SDS, 0.1 mg/ml BSA, 0.1 mg/ml Herring Sperm DNA. Next the slides were washed at 55°C with 2X SSC, 0.5% SDS for 1 minute with a 30 second soak and washed again at 45°C for 1 minute with the same solution. The resuspended labeled targets were injected into the chambers and hybridized for 16 hours at 45°C with sample agitation. The slides were then washed as follows: 2 times 1 minute 30 seconds at 45°C with 30 seconds soaking in 2X SSC, 0.5% SDS, 1 time 1 minute at 45°C in 2X SSC, 0.5% SDS, 2 times 1 minute 30 seconds at 45°C with 30 seconds soaking in 0.5X SSC, 0.1% SDS, 1 time 1 minute at 37°C with 20 seconds soaking in 0.5X SSC, 0.1% SDS, 1 time 1 minute at 23°C with 20 seconds soaking in 0.5X SSC, 0.1% SDS, 1 time 1 minute 30 seconds at 23°C with 30 seconds soaking in 0.1X SSC, 1 time 30 seconds at 23°C in 0.1X SSC and 2 times 30 seconds at 23°C in milliQ filtered water. Finally slides were dried for 2 minutes 30 seconds with nitrogen gaz. Slide scanning and image processing were performed as described in Table 6.

### Microarray data processing and analysis

A procedure was developed to process and analyze microarray intensity data from single dyes, as opposed to fold change methods routinely used for spotted arrays. Data analyses were performed using customized scripts for R and Bioconductor (http://www.r-project.org and http://www.bioconductor.org). Spots that were flagged as presenting abnormal morphology during the image processing were replaced by mean value of the remaining spots of the same probe from the other slides from the same sample type. Background intensities were subtracted from the foreground intensities. Background-subtracted data were log2-transformed and normalized using quantile correction approach.

A filtering step was applied to select positive genes to be used for further analysis. The mean intensity of spots containing buffer only was calculated for each row of sub-grids, and was taken as the minimum intensity of probes for that subgrid. A probe was called positive (detected above background) when its signal intensity was above the buffer intensity on at least 50% of slides within a given sample type. When determining differential expression, positive probes were probes that were detected according to this criterion in at least one of the tissues (e.g. phelloderm and xylem juvenile). Mean probe intensity was determined for genes represented by more than one positive probe. All microarray experiment data has been submitted to the Gene Expression Omnibus (GEO) under accession numbers GSE35624, GSE35847 and GSE35922.

Statistical testing for differential gene expression used the linear modeling approach and the empirical Bayes statistics [40]; the p-values were adjusted for multiple testing according to Benjamini and Hochberg [41]. Differential genes were those meeting an adjusted p-value ≤0.01, unless stated otherwise.

### The PiceaGenExpress database

The PiceaGenExpress database was developed from transcript profiles obtained for eight different tissue types coming from five independent experiments (see Table 6). For this, the average signal intensity was determined from all of the slides available for a sample type. The genes were then ranked based on their average signal intensities within a tissue type and equally divided into 10 separate classes according to their signal intensity. Genes from class 1 or class 10 were the 10% with lowest and highest signal intensities, respectively (for the number of genes per class in each type, see Additional file 6: Table S2 and Additional file 8: Figure S5). Functional annotations were based on the matches with Arabidopsis proteins (TAIR 9 release) with E-value <1e-10 and on the detection of Pfam domains as described [13]. PiceaGenExpress is made available as a flat file (Additional file 5: Table S1), which may be uploaded to any type of data processing or spreadsheet platform.

Agglomerative hierarchical clustering with the complete linkage method was performed using hclust function in R [42] on the expression levels for two gene families, i.e. dehydrins and LEA proteins. This approach used a similarity matrix based on Euclidean distance. A smaller distance means that two genes or clusters have more similar expression levels in the tissues analyzed. First, the clustering analysis placed each gene into its own singleton group or cluster. Second, the closest clusters were iteratively joined together until all genes were merged into a single cluster based upon similarity/distance measures between clusters. Dendrograms showing clusters of genes were drawn (Additional file 9: Figure S6).

### RNA-Seq data processing and analysis

Two composite samples were analysed by RNA- Sequencing for validation and comparison to MA results. Samples were prepared by combining equal molar amounts of the P. glauca RNAs isolated from secondary xylem (juvenile trees) and phelloderm. These samples were also used in the MA profiling of each of the tissues. RNA-Sequencing, filtering of quality reads and mapping of reads onto the cDNA clusters were described [13]. The number of sequences matching each cDNA cluster was normalized by transforming the data to the number of reads per kilobase of exon model per million mapped reads (RPKM) following the method of Mortazavi et al. [24]. Unless specified otherwise, an RPKM >1 was used as a minimum threshold of detection for RNA-Seq. Differential expression of genes was determined by using the Chi-squared test corrected for multiple testing according to Benjamini and Hochberg [41].

## Competing interests

The authors declare that they have no competing interests.

## Authors’ contributions

PR, BB, JB and JM participated in the microarray design; ER, BB, SC, CB, JM designed experiments; ER, SC, J-PV, IG prepared samples, extracted RNAs, and carried out hybridizations and data acquisition; ER, PR, P-LP, J-PV developed data analyses procedures and analysed the test array; ER and SC created the PiceaGenExpress database; SC submitted microarray design and data to public databases; ER, SC and JM drafted the manuscript. All of the authors approved the manuscript.

## Supplementary Material

Additional file 1 Additional material Experimental tests of optimal oligonucleotide design and manufacture methods. Click here for file

Additional file 2 **Figure S1.** Effect of SNPs on hybridization signal intensities and differential expression ratios. Hybridization data were based on five biological replications of each white spruce tissue tested, and two technical replicates (dye swaps) were used for each sample. Each data point represents the mean value for the five biological replicates. For probe intensities (A, C, E), the data are based on hybridizations with total RNA from secondary xylem; each point represents the mean value data for Alexa Fluor 555 (green) or Alexa Fluor 647 (red). The ratios (B, D, F) were obtained from pair-wise comparisons of secondary xylem and young needles; each dot represents the mean ratio obtained from the dye-swaps of all five biological replicates. Click here for file

Additional file 3 **Figure S2.** Comparison of differential expression results from a cDNA microarray and the test oligonucleotide microarray. Hybridization data were based on five biological replications of each white spruce tissue tested, and two technical replicates (dye swaps) were used for each sample. Tissue preferential expression was determined as described (Pavy et al. 2008) for secondary xylem and young needles. The outcomes of the two types of arrays were compared by assessing the presence or absence of statistically significant tissue preference. Click here for file

Additional file 4 **Figure S3.** Interspecific comparison of hybridization intensities in the phelloderm. A-F: Pair-wise comparison of white spruce and six other species based on the number of shared positive probes indicated in the plots. The squared correlation coefficients (r^2^) are as follows 0.83 (A), 0.84 (B), 0.90 (C), 0.18 (D), 0.30 (E) and 0.18 (F). G: Analysis of signal intensity variation between species; the fold change (FC) was determined from the average normalized signal intensities (log2 scale). An FC of 1 or −1 represents a two-fold signal increase or decrease, respectively. Click here for file

Additional file 5 **Table S1**. PiceaGenExpress transcript profiles. Click here for file

Additional file 6 **Table S2**. Summary statistics of Picea Gen Express transcript profiles. Click here for file

Additional file 7 **Figure S4.** Hybridization signal intensities of genes in each of the 10 expression classes in each tissue in PiceaGenExpress transcript profiles. Vegetative buds (A), megagametophytes (B), xylem from mature trees (C), phelloderm from juvenile trees (D), xylem from juvenile trees (E), embryogenic cells (F), needles (G) and roots (H). RPKM values from RNA-sequencing of phelloderm (I) and xylem (J) of juvenile trees are also presented. Click here for file

Additional file 8 **Figure S5.** Expression classes and numbers of tissue of annotated and non annotated sequences. A-E: Number of annotated and non annotated sequences per expression class for embryogenic cells (A), megagametophytes (B), xylem from mature trees (C), phelloderm (D) and vegetative buds (E). F: Number of tissues in which each annotated and non-annotated sequence was detected. Frequency, number of genes in a given intensity class or detected in a given number of tissues types. Click here for file

Additional file 9 **Figure S6.** Hierarchical clustering dendrograms of gene expression within two osmotic regulation protein families: A) dehydrins, and B) late embryogenesis abundant (LEA) proteins. Each leaf node of the dendrograms corresponds to an individual gene, and each node (horizontal line) represents a gene cluster. A gene cluster is composed of individual genes or existing gene cluster with the fusion point. Each gene cluster was placed at a height level as shown on the vertical axis. Height values refer to the similarity/distance measures between genes and gene clusters. Click here for file
