# Supplementary material for: Transcriptome profiling in conifers and the PiceaGenExpress database show patterns of diversification within gene families and interspecific conservation in vascular gene expression
Source: BMC Genomics. 2012 Aug 29;13:434. doi: 10.1186/1471-2164-13-434 (PMC3534630; doi:10.1186/1471-2164-13-434)
Supplement: Additional file 1 — Additional material Experimental tests of optimal oligonucleotide design and manufacture methods. [file 1471-2164-13-434-S1.pdf]

Supplemental Materials 1.

Title: Experimental tests of optimal oligonucleotide design and manufacture methods.

Introduction

A test microarray was developed and utilized to assess the impacts of oligonucleotide design parameters and manufacturing methods. The key parameters that were tested are oligonucleotide probe length (affecting Tm), the impact of SNPs at different positions on the oligonucleotide, impact of insertions or deletions of up to 6 nucleotides. The manufacturing characteristics were those expected to affect spot morphology and signal quality such as slide surface chemistry and spotting buffer. The results reported here are from a tissue comparison experiment was carried out with RNAs isolated from needles (foliage) and secondary xylem of young white spruce trees (P. glauca) that previously studied by using an 11k cDNA microarray [1].

Materials and methods

Genes used for test chip development

A set of 1,000 cDNAs were selected based upon differential expression between needles and xylem according to Pavy et al. [1], on their of expression level secondary xylem in mature trees (this report). See Table 1. The sequences were mapped to 929 cDNA clusters or full-length cDNA described in *P. glauca* [2]. The full-length cDNAs or the longest representative cDNA clone from each cluster were used to design and analyse the oligonucleotide probes. In addition, 96 of the cDNAs were also amplified by PCR as described in Pavy et al. [1] and amplicons were spotted along side with the oligonucleotides (Table 1).

Oligonucleotide design parameters

Variations of probe designs and control probes were as follows:

- Perfect match probes 70-mer oligonucleotides probes (1093 probes, representing 929 cDNAs) with one or two probe per gene. These probes were used as reference probes.
- Truncated probes: one 60-mer oligonucleotide and one 50-mer oligonucleotide was designed to overlap with the 70-mer reference probe for comparison purposes, for 50 genes.

- Sets of robes with equidistant SNPT mismatches distributed throughout the oligonucleotide (N=1, 3,7 , 10, 14, 21) (%id=98,95,90,85,80,70), for 50 genes.
- Probes with mismatching ends: 70-mers designed to match with the corresponding reference probe over 60 or and 50 nucleotides and dangling ends, for 50 genes.
- Sets of probes with insertions and deletions of (1,2,3,6) nucleotides in the middle of a probe, for 50 genes.
- Position controls (50 genes): probes located in the 3', middle and 5' parts of the same gene, as defined by 500-base intervals
- Synthesis controls (50 probes): same oligo synthesized twice
- Negative controls (50 alien control probes)

Microarray manufacture

The oligonucleotides were obtained from Invitrogen (Carlsbad, CA, USA). The microarray printing was carried out at the Genome BC Microarray Platform (Vancouver, BC, Canada) using 4 x 12 spot sub-arrays. Glass slides with two types of surface chemistry were tested and compared: aminosilane coated slides (Erie, Hudson, NH, USA) and epoxysilane coated slides (Erie, Hudson, NH, USA). In addition, variations of the spotting buffer were used to resuspend the oligonucleotides: 3x SSC or 3x SSC with 1M betaine spotted side-by-side on the microarray.

Biological material

For this experiment, 2 year-old white spruce seedlings produced from open-pollinated seed lots were obtained from a commercial nursery (CPPFQ nursery, Ste-Anne de Beaupré, QC, Canada). Seedlings were transferred to 8” diameter pots, and allowed to grow under greenhouse conditions with standard watering and fertilizing as applied for optimal growth, but no artificial lighting or heating. After eight weeks, most of the plants had completed their primary growth for the season and formed a small terminal but. At that time, young needles (formed during the same season) and secondary xylem tissue were collected from the main stem of 30 trees. To minimize uncontrolled variability between samples, individual samples were randomly mixed to form pools of five trees. Each pool was considered as a biological replicate, and five replicates were analysed for each tissue. Total RNA was extracted as described in Pavy et al. [1].

RNA amplification and labelling

One µg of total RNA was amplified for each replicate with the Amino Allyl MessageAmp II aRNA Amplification Kit (Applied Biosystems by Life Technologies, Carlsbad, CA, USA) following manufacturer's instructions. Five µg of amplified RNA (aRNA) was then labelled with Alexa Fluor 555 or 647 dyes (Invitrogen, Carlsbad, CA, USA) and purified as described in the manual. Incorporation efficiency of the dyes was evaluated using a Nanodrop spectrophotometer (Thermo Fisher Scientific, Waltham, MA, USA). The two samples to be hybridized together were mixed and volume was reduced to ~10µl in a DNA 120 speedvac (Thermo Fisher Scientific, Waltham, MA, USA). Labelled aRNAs were fragmented for 15 minutes at 70°C using Ambion's «RNA Fragmentation Reagents» (Applied Biosystems by Life Technologies, Carlsbad, CA, USA), placed on ice for 1 minute, denatured for 2 minutes at 95°C, put on ice for 2 min and resuspended in 120 µl hybridization buffer preheated to 55°C. Samples were kept in a heating block at 46°C until hybridization. Hybridizations were performed in HS400Pro hybridization stations (Tecan Group Ltd., Männedorf, Switzerland).

Results

Assessment of microarray manufacture methods

When processed with our standard hybridization protocol (see materials & methods), aminosilane coated slides were clean, with low background, high spot signal intensity and good spot morphology. On the other hand, epoxysilane coated slides presented high background, low spot intensity and significant spots bleeding all over. We also tested the addition of betaine to the 3X SSC spotting buffer to reduce the occurrence of donut-shaped spots. We observed that the addition of betaine had an unexpected effect: spots which contained betaine showed a systematical effect of "bleeding" causing the spots appear as long streaks. We concluded that the optimum conditions were obtained by using aminosilane coated slides and 3X SSC without betaine as a spotting buffer.

Impacts of probe design.

The impact of probe design parameters was assessed in terms of probe specificity and assay sensitivity based on hybridization signal intensities (Supp. Fig. 1A, C, E), differential transcript accumulation ratios when comparing different tissues (secondary xylem and phloem, see

methods)(Supp. Fig. 1B, D, F), and detection of differential transcript accumulation with oligonucleotide probes compared to cDNA amplicons (Supp. Fig. 2). The data presented here are primarily for 70-mer oligonucleotides unless specified as being different.

The presence of single nucleotide polymorphisms (SNPs) had a most strong and consistent effect on the microarray hybridization outcomes. These findings were based on signal intensities and ratios and by comparing probes with SNPs to reference probes with no mismatches. Figure 1 shows that a single SNP mismatch in a 70-mer probe had a small effect on probe intensities (Supp. Fig. 1A). Similarly, the ratio of expression when comparing two tissues, secondary xylem and young needles in the present study, was largely unaffected (Supp. Fig. 1B). Because the SNP was positioned at the center of the oligonucleotide probe, its impact on hybridization intensity was assumed to be maximal, as SNPs that are closer to one end of the probe would result in longer stretches of perfect match sequences. We were able to infer that SNP near the end of the probes would have very little impact, because we observed that 70-mer probes with degenerate ends gave results that were comparable to the reference (not shown).

Probes were also designed with 3, 7, 10, 14, or 21 SNP mismatches, representing 95% to 70% sequence identity. In each case the SNPs were equidistant from one another and distributed throughout the probe. The presence of three SNP mismatches (distributed throughout the oligonucleotide probes) had a small to moderate effect on hybridization signal intensities (Supp. Fig. 1C) and in many cases the expression ratio was not largely altered (Supp. Fig. 1D). In contrast, the presence of seven SNP mismatches in the probe had a large effect on intensities and expression ratios (Supp. Fig. 1E, F). Based on these data, 70-mer probes that vary by up to three SNPs distributed throughout the probe (95.7% sequence identity) are expected to hybridize to the same RNA and on average produce a signal of similar strength. Probes with seven SNPs or more, on the other hand, give very little cross hybridization. These observations establish a threshold of sensitivity to sequence variation and for specificity with a cut-off between four and six SNPs, i.e. between 95% and 91%.

These findings on the impacts of SNPs were compared to independently obtained results for the same genes based on analyses using an amplicons cDNA microarray and comparing the same tissue samples as those analysed here from secondary xylem and young needles [1]. A total of 93%

probes with no mismatches gave the same differential expression response (significant DE at $p<0.01$, with correction for multiple testing) as found for the same gene by using cDNA microarray (Supp. Fig. 2). The presences of 1 or 3 SNP mismatches decreased the agreement of results slightly to 87% and 78% respectively. In contrast, almost no agreement was found with 7 SNPs or more.

The presence of single insertion or deletion of 1, 2, 3, or 6 nucleotides positioned at the center of the probe had little effect on the microarray hybridization outcomes. Supp. Fig. 2 show that the despite these insertions and deletions, tissue preferential expression was highly congruent with the cDNA array results (85%-90% of genes) found in Pavy et al. [1].

Both SNPs and indels are frequent in many conifer coding sequences and represented potential sources of variation that were expected to influence the outcomes of microarray in spruce coding sequences. The data obtained from these probe comparisons are sufficient to model their effects and minimize their impacts in global probe design and microarray experiments.

The other factors we examined had minor and less predictable effects on hybridization outcomes. There was little variation across different high quality 70-mer probes designed at different position within the same gene. The variation appeared random and had a minor impact on the detection of differential expression; the data did not suggest a significant bias of probes located near the 3' or the 5' of the cDNA sequence (not shown). The different probe lengths (50-mer, 60-mer and 70-mer) also had an effect on hybridization signal intensities. As might be expected, the 60-mer and 70-mer oligonucleotides gave slightly more robust results and generally produced stronger hybridization signals than the 50-mers.

References

1. Pavy, N, Boyle, B, Nelson, et al. (2008) Identification of conserved core xylem gene sets: conifer cDNA microarray development, transcript profiling and computational analyses. *New Phytol.* 180: 766-786
2. Rigault P, Boyle B, Lepage P, Cooke J, Bousquet J, MacKay J (2011) A white spruce gene catalogue for conifer genome analyses. *Plant Physiol.* 157: 14-28

Tables and Figures

Table 1. Expression of cDNA selected for test microarray.

	Tissue preferential transcript accumulation			Intensity of transcripts detected in secondary xylem of mature trees			
Sequences selected from cDNA microarray studies	Needle	Xylem	None	Total	Strong	Moderate	Low
Total number of cDNAs selected	295	295	410	512	109	126	277
%	30%	30%	41%	51%	21%	25%	54%
cDNA represented by an amplicon on test microarray	29	29	38	50	11	12	27

Figure Legends

Supp. Fig. 1. Effect of SNPs on hybridization signal intensities and differential expression ratios. Hybridization data were based on five biological replications of each white spruce tissues tested, and two technical replicates (dye swaps) were used for each sample. Each data point represents the mean value for the five biological replicates. For probe intensities (A, C, E), the data are based on hybridizations with total RNA from secondary xylem; each point represents the mean value data for Alexa Fluor 555 (green) or Alexa Fluor 647 (red). The ratios (B, D, F) were obtained from pair-wise comparisons of secondary xylem and yound needles; each dot represents the mean ratio obtained from the dye-swaps of all five biological replicates.

Supp. Fig, 2. Comparison of differential expression results from a cDNA microarray and the test oligonucleotide microarray. Hybridization data were based on five biological replications of each white spruce tissues tested, and two technical replicates (dye swaps) were used for each sample. Tissue preferential expression was determined as described [1] for secondary xylem and young needles. The outcomes of the two types of arrays were compared by assessing the presence or absence of statistically significant tissue preference.
