# Supplementary material for: Transcriptome profiling in conifers and the PiceaGenExpress database show patterns of diversification within gene families and interspecific conservation in vascular gene expression
Source: BMC Genomics. 2012 Aug 29;13:434. doi: 10.1186/1471-2164-13-434 (PMC3534630; doi:10.1186/1471-2164-13-434)
Supplement: Additional file 2 — Figure S1. Effect of SNPs on hybridization signal intensities and differential expression ratios. Hybridization data were based on five biological replications of each white spruce tissue tested, and two technical replicates (dye swaps) were used for each sample. Each data point represents the mean value for the five biological replicates. For probe intensities (A, C, E), the data are based on hybridizations with total RNA from secondary xylem; each point represents the mean value data for Alexa Fluor 555 (green) or Alexa Fluor 647 (red). The ratios (B, D, F) were obtained from pair-wise comparisons of secondary xylem and young needles; each dot represents the mean ratio obtained from the dye-swaps of all five biological replicates. [file 1471-2164-13-434-S2.pdf]

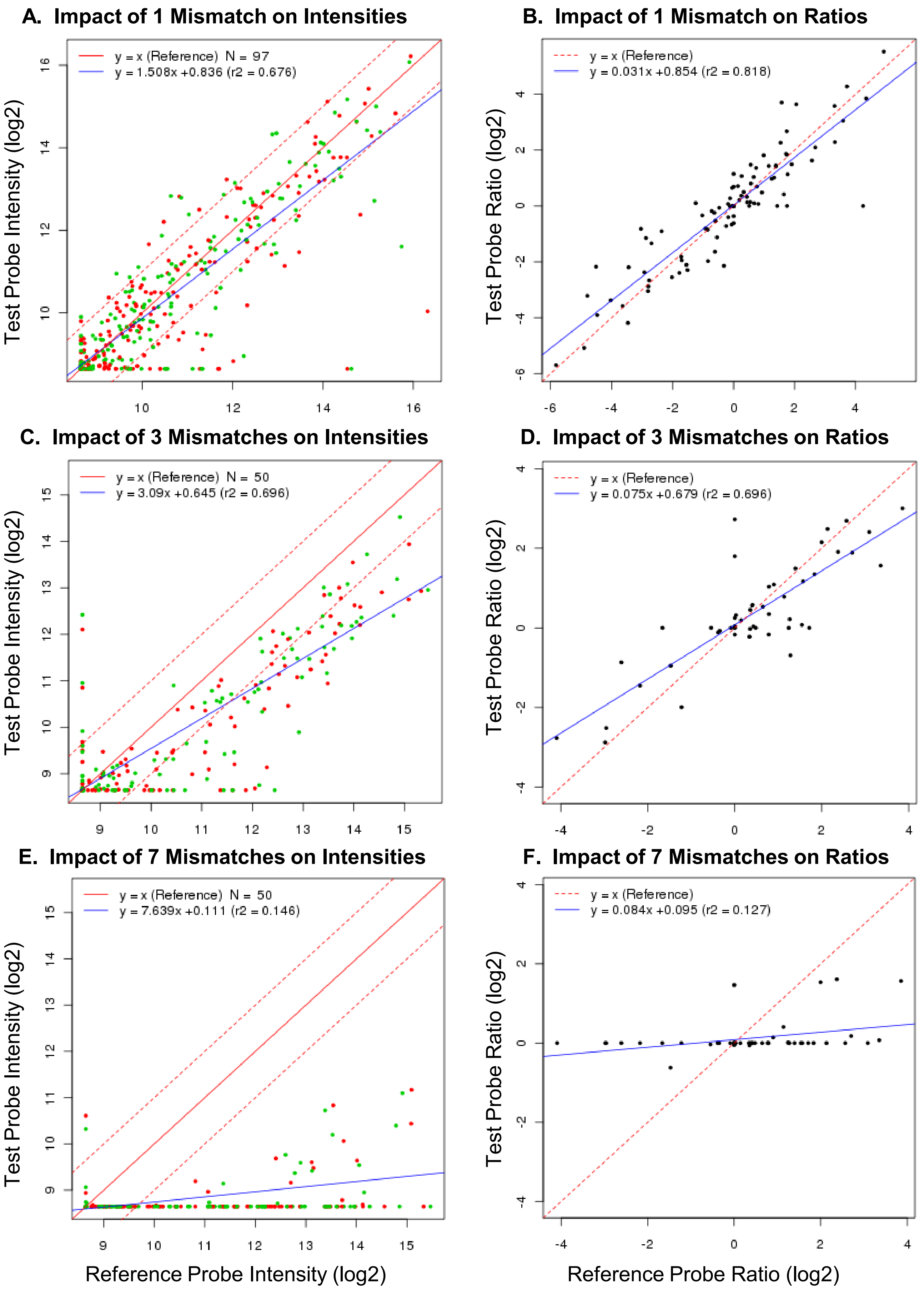

Additional figure 1
