# Supplementary figures and images for: Transcriptome profiling in conifers and the PiceaGenExpress database show patterns of diversification within gene families and interspecific conservation in vascular gene expression
Source: BMC Genomics. 2012 Aug 29;13:434. doi: 10.1186/1471-2164-13-434 (PMC3534630; doi:10.1186/1471-2164-13-434)

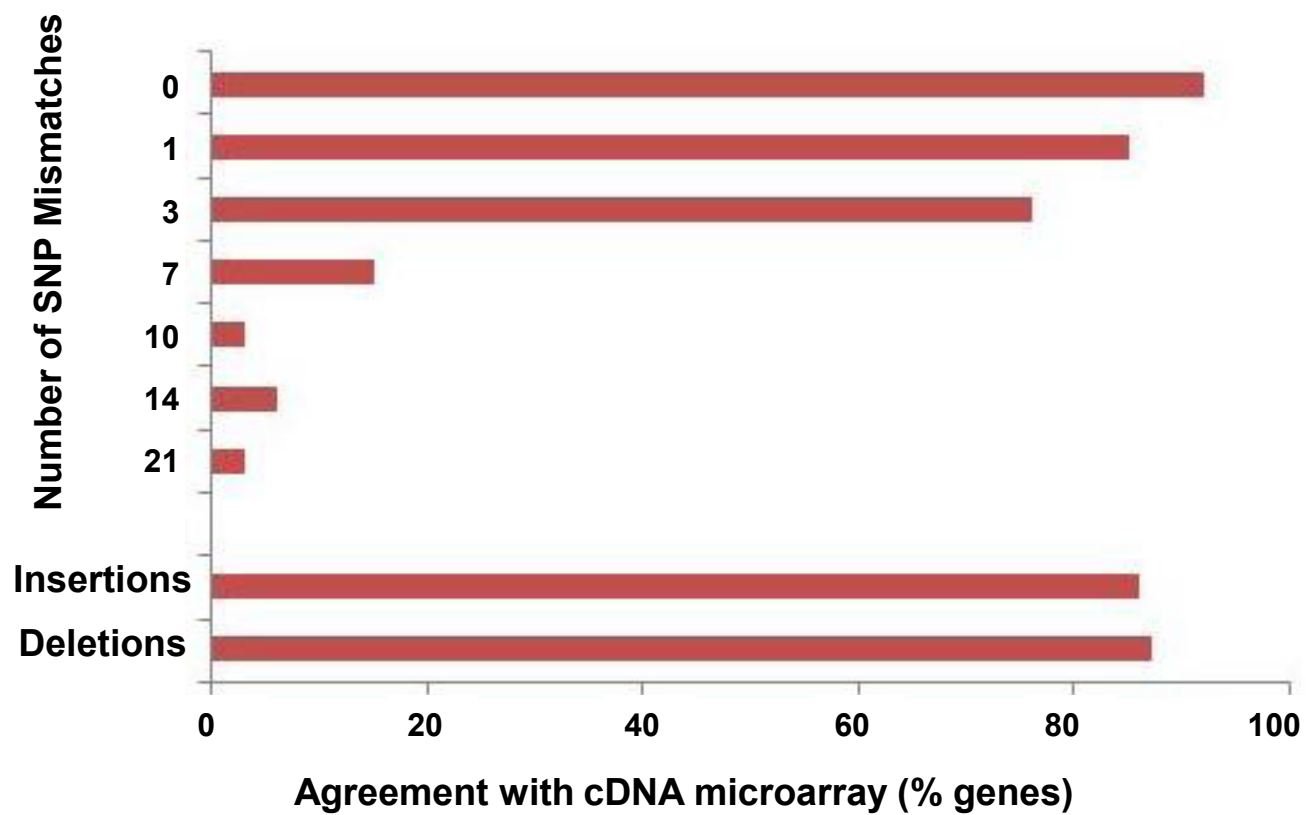

Additional figure 2

Supplement: Additional file 3 — Figure S2. Comparison of differential expression results from a cDNA microarray and the test oligonucleotide microarray. Hybridization data were based on five biological replications of each white spruce tissue tested, and two technical replicates (dye swaps) were used for each sample. Tissue preferential expression was determined as described (Pavy et al. 2008) for secondary xylem and young needles. The outcomes of the two types of arrays were compared by assessing the presence or absence of statistically significant tissue preference. [file 1471-2164-13-434-S3.pdf]

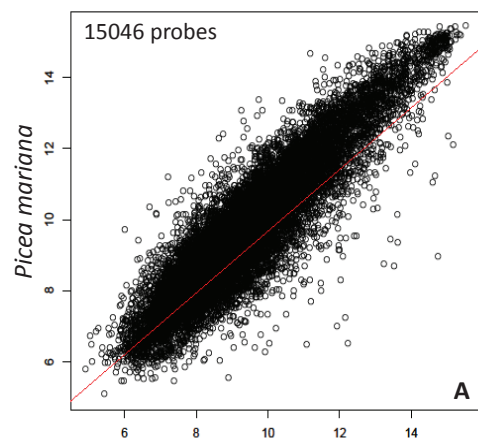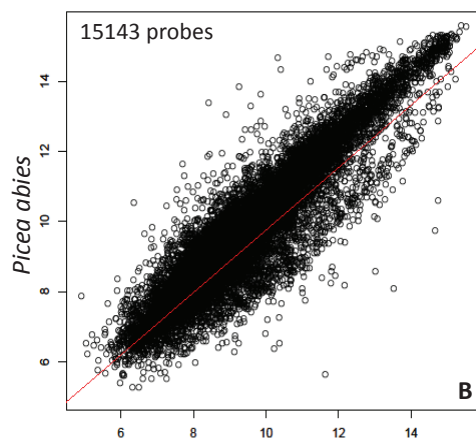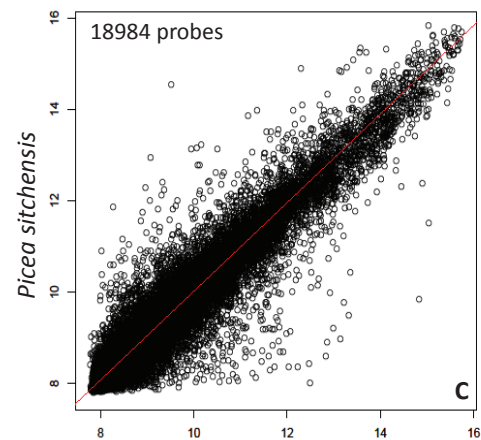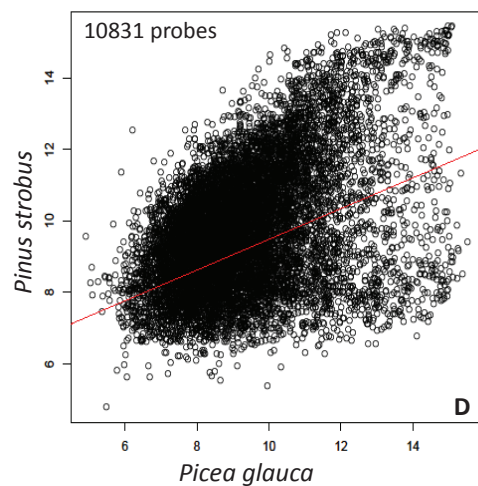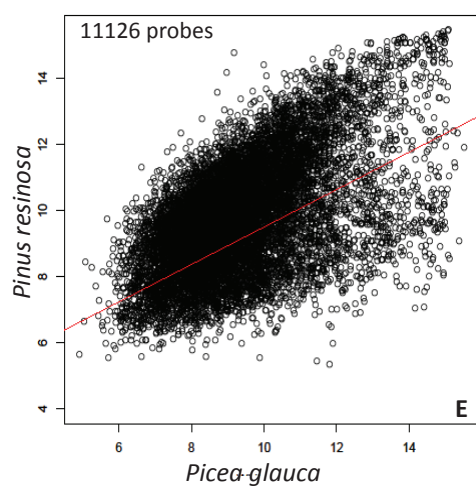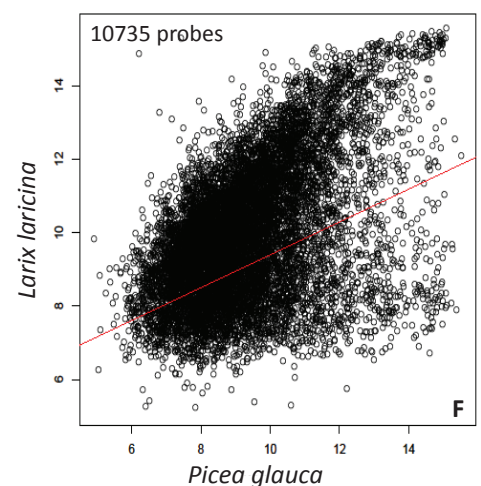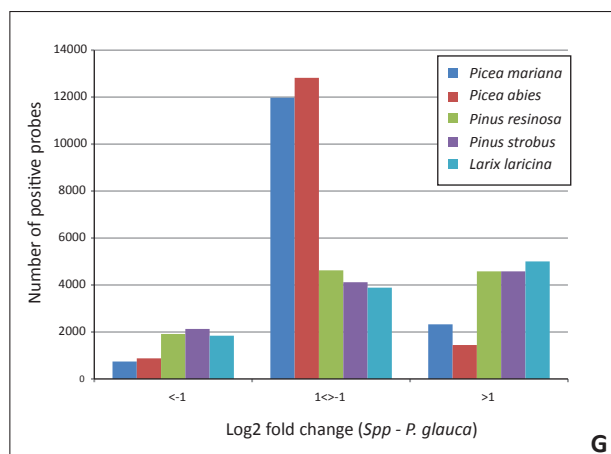

Additional figure 3

Supplement: Additional file 4 — Figure S3. Interspecific comparison of hybridization intensities in the phelloderm. A-F: Pair-wise comparison of white spruce and six other species based on the number of shared positive probes indicated in the plots. The squared correlation coefficients (r2) are as follows 0.83 (A), 0.84 (B), 0.90 (C), 0.18 (D), 0.30 (E) and 0.18 (F). G: Analysis of signal intensity variation between species; the fold change (FC) was determined from the average normalized signal intensities (log2 scale). An FC of 1 or −1 represents a two-fold signal increase or decrease, respectively. [file 1471-2164-13-434-S4.pdf]

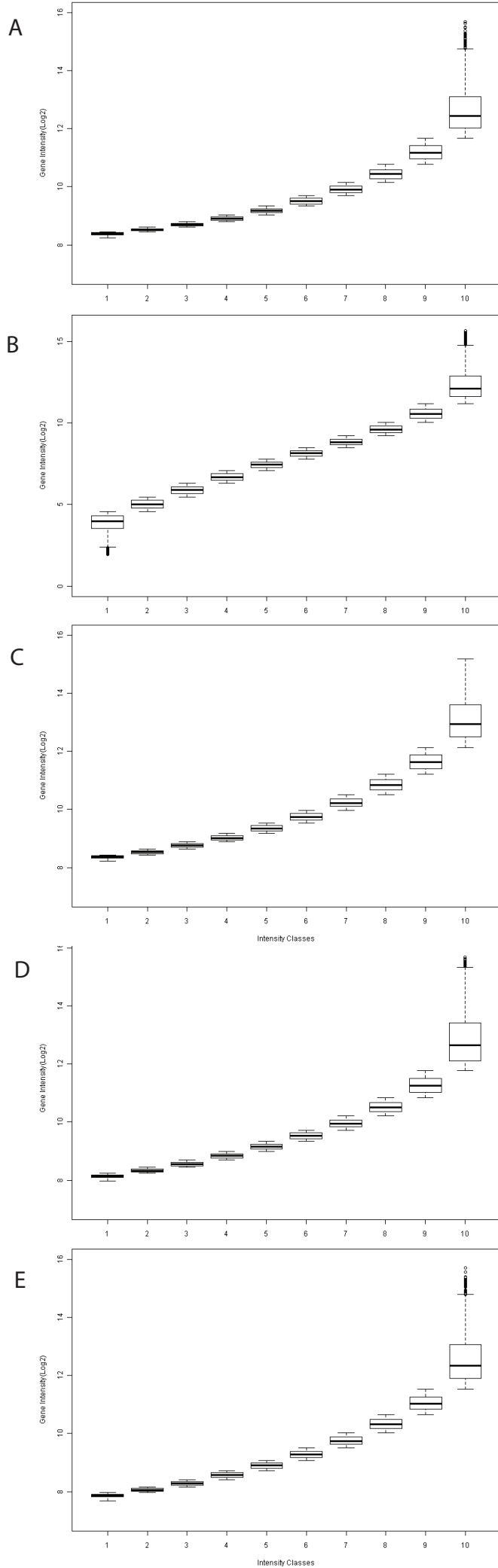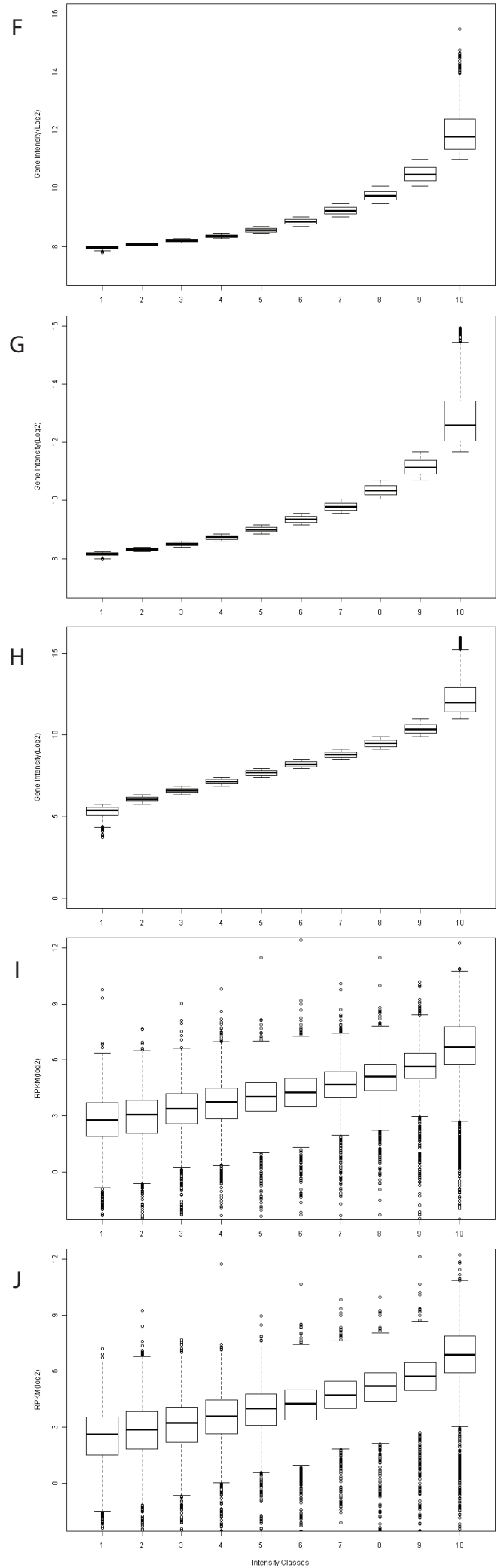

Additional figure 4

Supplement: Additional file 7 — Figure S4. Hybridization signal intensities of genes in each of the 10 expression classes in each tissue in PiceaGenExpress transcript profiles. Vegetative buds (A), megagametophytes (B), xylem from mature trees (C), phelloderm from juvenile trees (D), xylem from juvenile trees (E), embryogenic cells (F), needles (G) and roots (H). RPKM values from RNA-sequencing of phelloderm (I) and xylem (J) of juvenile trees are also presented. [file 1471-2164-13-434-S7.pdf]

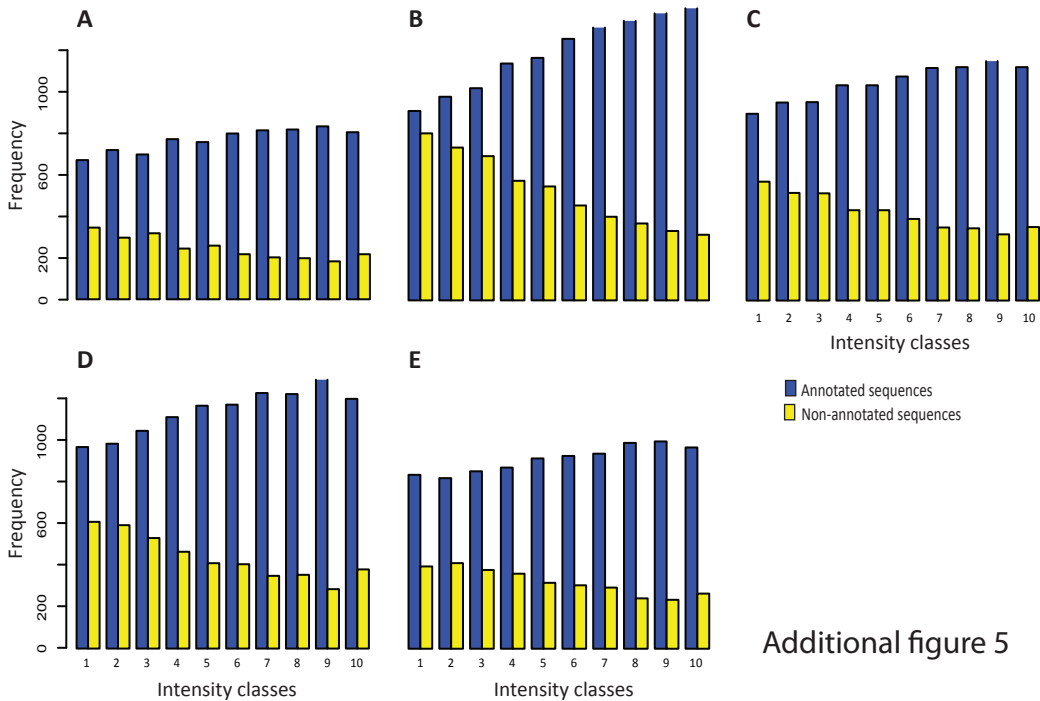

Additional figure 5

Supplement: Additional file 8 — Figure S5. Expression classes and numbers of tissue of annotated and non annotated sequences. A-E: Number of annotated and non annotated sequences per expression class for embryogenic cells (A), megagametophytes (B), xylem from mature trees (C), phelloderm (D) and vegetative buds (E). F: Number of tissues in which each annotated and non-annotated sequence was detected. Frequency, number of genes in a given intensity class or detected in a given number of tissues types. [file 1471-2164-13-434-S8.pdf]

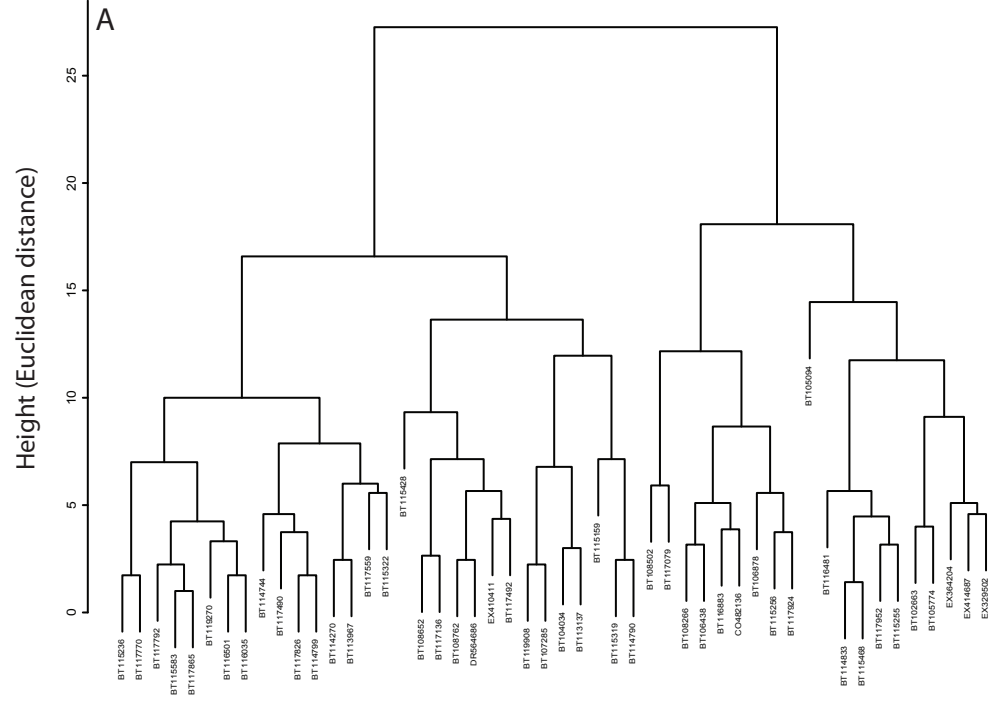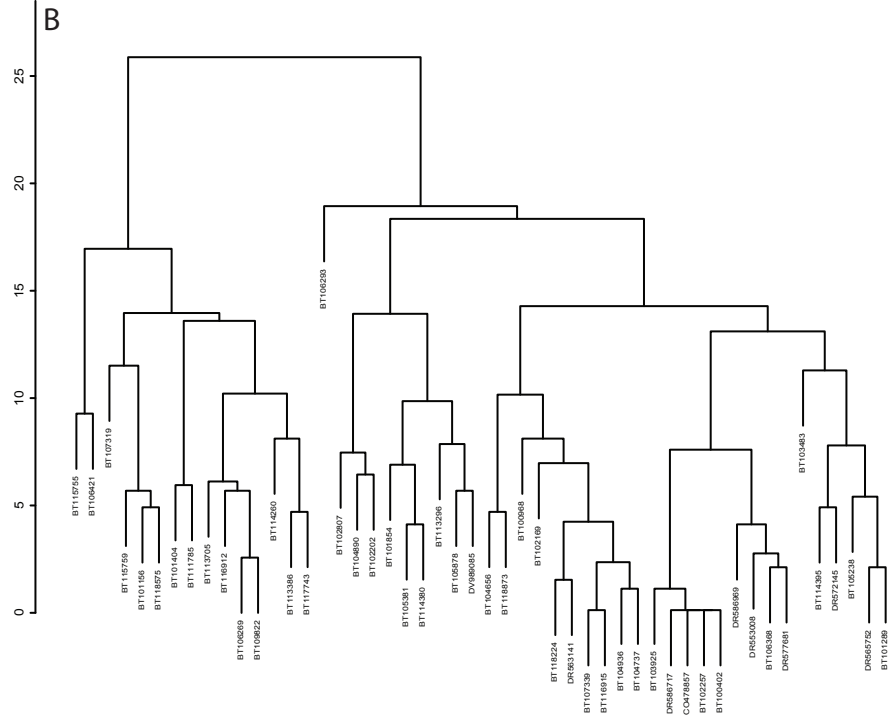

Supplement: Additional file 9 — Figure S6. Hierarchical clustering dendrograms of gene expression within two osmotic regulation protein families: A) dehydrins, and B) late embryogenesis abundant (LEA) proteins. Each leaf node of the dendrograms corresponds to an individual gene, and each node (horizontal line) represents a gene cluster. A gene cluster is composed of individual genes or existing gene cluster with the fusion point. Each gene cluster was placed at a height level as shown on the vertical axis. Height values refer to the similarity/distance measures between genes and gene clusters. [file 1471-2164-13-434-S9.pdf]
